# Differentiations of determinants for the community compositions of bacteria, fungi, and nitrogen fixers in various steppes

**DOI:** 10.1002/ece3.4940

**Published:** 2019-02-22

**Authors:** Rong Sheng, Ke Li, Wenzhao Zhang, Hai Wang, Honglin Liu, Xiaoya Zhu, Hongxin Wu, Xiaoqing Zhang, Qimei Lin, Xuecheng Sun, Yafang Tang, Lamus A, Wenxue Wei

**Affiliations:** ^1^ Key Laboratory of Agro‐ecological Processes in Subtropical Regions and Taoyuan Agro‐ecosystem Research Station, Soil Molecular Ecology Section, Institute of Subtropical Agriculture Chinese Academy of Sciences Changsha China; ^2^ Institute of Grassland Science Chinese Academy of Agricultural Sciences Huhehaote China; ^3^ College of Resources and Environmental Sciences China Agricultural University Beijing China; ^4^ College of Resources and Environmental Sciences Huazhong Agricultural University Wuhan China; ^5^ Hubei Key Laboratory of Quality Control of Characteristic Fruits and Vegetables, College of Life Science and Technology Hubei Engineering University Xiaogan China

**Keywords:** bacteria, community composition, diazotroph, fungi, steppe grassland

## Abstract

1. Different types of steppes could provide heterogeneous habitat environments for underground microorganisms, but much less is known about how soil microbes fit the distinct habitats and what are the underlying mechanisms in shaping their community patterns.
2. We simultaneously examined the community compositions and structures of soil bacteria, fungi, and diazotrophs across desert, typical, and meadow steppes in Inner Mongolia using high‐throughput sequencing.
3. The results showed that soil bacteria, fungi, and diazotrophs exhibited different distribution patterns across steppe types. Although different steppes displayed obvious differences in climate conditions, plant traits, and soil properties, most of bacterial species were shared by all the steppes while only a few species were unique, indicating that the soil bacterial compositions were hardly influenced by the steppe types. Nevertheless, the habitat heterogeneity could cause shifts in the relative abundance of some bacterial groups, which resulted in significant changes in the community structure of soil bacteria across steppes. However, the fungal community compositions and structures were similar in typical and meadow steppes but that in desert steppe were significantly different. Whereas, the community compositions and structures of diazotrophs were strongly related to the steppe types. In this study, the similar parent material backgrounds of the steppe soils might be the important factor in shaping the homologous bacterial compositions. However, the variations in soil fertility, soil water repellency, and plant species across steppes would be the major driving forces in regulating the compositions and structures of fungal communities, while the diazotrophic communities would be more closely related to the changes in plant traits and soil fertility among steppes.
4. Our results provided evidence of habitat specificity for different microbial groups and their underlying drivers.

## 1 INTRODUCTION

Natural grassland covers about 400 million ha in China, comprising 41.7% of the total land area, which are mainly distributed in Inner Mongolia and Tibetan Plateau (Kang, Han, & Sun, 2007). In Inner Mongolia, the grassland types change from meadow steppe to typical steppe and to desert steppe as mean annual precipitation decreases but temperature increases, from northeast to southwest, and precipitation is reported to be the primary factors in shaping various steppe types in this arid and semi‐arid grassland ecosystems (Ni & Zhang, 2000; Yan et al., 2015; Zhang et al., 2014). In these natural steppe ecosystems, soil microbes are important players in nutrient transformation, which would be crucial for supporting grass diversity and productivity (Van Der Heijden, Bardgett, & van Straalen, 2008). However, we have very limited knowledge about the underground microbial communities in relation to the steppe ecosystems.

It was strongly suggested that microbial assembly displays nonrandom environmental distributions (Fierer & Ladau, 2012; Hanson, Fuhrman, & Martiny, 2012; Hazard et al., 2013) and most microorganisms have specific habitat requirements (Bardgett et al., 2005; Lozupone & Knight, 2007). The picture that emerges from the existing literature is that microbial communities are subjected to many external structuring influences, including climate conditions (Pellissier et al., 2014; Wang et al., 2018), soil characters (Hu et al., 2014; Singh, Munro, Potts, & Millard, 2007), vegetations (Prober et al., 2015), and human activities etc (Degrune et al., 2017; Sheng et al., 2013). Different types of steppes possess distinctive characteristics of plant species composition, richness, and productivity (Yan et al., 2015; Zhang et al., 2014), which in turn lead to clear different livestock density and grazing intensity. All these differences may create obviously diverse habitat environments for below‐ground soil microbes (King, Farrer, & Schmidt, 2012; King et al., 2010). But it is not clear about the distribution patterns of soil microbes and the major driving factors in these natural ecosystems.

Till now, the biogeographic distribution of soil bacteria has been widely investigated, and soil pH is generally recognized as the major driving factor in continental ecosystems (Griffiths et al., 2011; Lauber, Hamady, & Fierer, 2009). However, there is a great diversity of underground microorganisms in steppe ecosystems, including bacteria, fungi, archea, protest, etc. Furthermore, there are also various functional groups that regulated specific soil functions. It is not clear whether all these microorganisms in soils behave similarly or differently as bacteria. It has been reported that soil fungi, bacteria, and functional groups showed significant differences in niche types (Tedersoo & Bahram, 2016), and the community assemblage processes may also differ between different microbial groups. For example, compared with bacteria, fungi are more capable of decomposing recalcitrant organic materials, adapting to soil conditions of low nitrogen and high C:N ratio, and tolerating acidic soils (Klein, Swinnen, Thevelein, & Nevoiqt, 2017; Rousk, Brookes, & Bååth, 2009; Strickland & Rousk, 2010). Fungal community changed more during the ecosystem succession than bacteria (Zhong, Yan, Wang, Wang, & Shangguan, 2018). Besides, some functional microorganisms, such as nitrogen cycling, may behave differently because their community sizes are much smaller and more labile compared to the overall community of bacteria and fungi in soils (Henry, Bru, & Philippot, 2006; Stone, Kan, & Plante, 2015). Although different microbial groups exhibit significant differences in morphology, physiology, and biochemistry, they coexist in soil environment to sustain the soil function (Zheng et al., 2017). Complex networks of potential interactions generally occur among microbial communities, such as predation, competition, and mutualisms (Filion, St‐Arnaud, & Fortin, 1999; Rudnick, van Veen, & de Boer, 2015). However, the lack of simultaneously investigations of different soil microbial communities in steppe ecosystems restricts our understanding about the interactions between underground microorganisms and aboveground habitat types.

In this study, we conducted a 2,000 km transect soil sampling across three different steppes in Inner Mongolian, including desert, typical, and meadow grasslands. The compositions and structures of soil bacterial, fungal, and diazotrophic communities were simultaneously studied using high‐throughput sequencing technologies. Choosing diazotrophic community as an example of functional groups is due to the soils in natural steppe ecosystems are generally nitrogen limited and diazotrophs would be crucially important for the sustainability of the steppes (Dart & Wani, 1982; Kennedy & Islam, 2001). The objective is to explore the distribution patterns and the determining factors of bacteria, fungi, and diazotrophs in the various steppes and understand the possible mechanisms of soil microbial community formation in response to the changes in habitat environments of steppes.

## 2 MATERIALS AND METHODS

### 2.1 Soil sampling

Soil samples were collected in July 2017; the sampling sites were distributed from Huhhot (N 41°; E 111°) to Manzhouli (N 49°; E 119°), with an altitude from 592 to 1,447 m, covering three main types of grassland habitats including desert steppe, typical steppe, and meadow steppe (Supporting Information Figure S1). Along the 2,000 km transect, desert steppe soils are mainly collected in Siziwang and Sonid Right banners, with MAT and MAP of about 3.34°C and 186 mm, respectively. Typical steppe soil samples were taken from a wide region spans from Abaga banner (N43°54′; E115°20′) to Wulagai (N46°08′; E119°13′) with MAT and MAP of about 0.27°C and 307 mm, respectively. Meadow steppe soils were mainly sampled from Hulunbuir grassland with an approximately 100,000 km^2^ area in Inner Mongolia with MAT and MAP of about −1.93°C and 352 mm, respectively. The soils in these sampling regions are mainly derived from granite (Supporting Information Table S1). Totally 15 sampling sites were selected based on steppe types and distance; any two sampling sites were at least 50 kilometers apart. For each steppe type, five sampling sites were selected, respectively. At each sampling site, three soil samples were collected with about 50 m apart, and each sample of 1 kg surface soil (0–15 cm) was the mixture of five columns that randomly taken by a soil sampler. The sample was then divided into two portions; one (100 g) was packed into a sterile plastic bag and transported to the laboratory on ice within two days and archived at −80°C prior to molecular analyses. The remaining was air‐dried and subsequently used for the analysis of soil physiochemical properties. At the same time, grass community features, including species name and coverage, were surveyed for each plot. Coverage of individual species (plant coverage) was estimated using 1 × 1 m square.

### 2.2 Soil properties

Soil organic carbon (SOC) was determined by K_2_Cr_2_O_7_ oxidation (Kalembas & Jenkinso, 1973). Total nitrogen (TN) was measured with Automatic Flow Injection after digestion in H_2_SO_4_. Inductively coupled plasma spectrometry (ICP) was used to measure the total phosphorus (TP) and potassium (TK) after fusion in NaOH. Atomic absorption spectroscopy (AAS) was used to determine available K (AK) after extraction with 1 M CH_3_COONH_4_, while available P (AP) was measured following extraction with 0.5 M NaHCO_3_. Available nitrogen (AN) was measured using alkaline hydrolysis diffusion method (Bao, 2000). Soil pH was determined at a soil to water ratio of 1:2.5 (Bao, 2000). The water repellency was estimated with the WDPT test, conducted in the laboratory under controlled conditions (Tillman, Scotter, & Clothier, 1989). The soil properties are listed in Table 1.

**Table 1:** Climate condition, soil characteristics, and plant trait of different steppe types in the Inner Mongolia grassland

	Steppe types		
	Desert steppe	Typical steppe	Meadow steppe
WR(S)	22.5 ± 7.69 a	6.31 ± 1.18 b	7.73 ± 0.61 b
SOM (g/kg)	0.89 ± 0.61 b	3.68 ± 2.08 a	3.61 ± 1.57 a
TN (g/kg)	0.07 ± 0.04 b	0.24 ± 0.12 a	0.21 ± 0.07 a
AN (mg/kg)	26.8 ± 17.81 b	93.17 ± 47.10 a	106 ± 32.8 a
DOC (mg/kg)	0.32 ± 0.11 b	0.54 ± 0.15 a	0.33 ± 0.04 b
TK (g/kg)	2.52 ± 0.11 a	2.58 ± 0.15 a	2.62 ± 0.13 a
AK (mg/kg)	0.3 ± 0.05 a	0.29 ± 0.12 a	0.35 ± 0.1 a
TP (g/kg)	0.02 ± 0.01 a	0.03 ± 0.01 a	0.04 ± 0.01 a
AP (mg/kg)	9.93 ± 5 a	9.30 ± 0.12 a	10.34 ± 1.44 a
PH (1:2.5)	7.22 ± 0.24 a	7.21 ± 0.33 a	6.9 ± 0.1 a
MAT (°C)	3.34	0.27	−1.93
MAP (mm)	205	302	351
PC (%)	19 ± 12.45 b	57 ± 19.87 a	60 ± 11.73 a
PR*	9.9 ± 0.2	13.3 ± 0.3	19.4 ± 0.5
PD*	9.7 ± 0.3	14.7 ± 0.5	27.3 ± 0.2

Notes

Values represent *M* ± *SD* (*n* = 5). ANOVA was used to test the differences between steppe types. Different letters (a, b) in the same row represent significant differences (*p* < 0.05).

AK: Available potassium; AN: available nitrogen; AP: Available phosphorus; DOC: dissolved organic carbon; MAP: Mean annual precipitation; MAT: Mean annual temperature; PC: Plant coverage; PD: Plant beta‐diversity; PR: Plant richness; SOM: Soil organic matter; TK: Total potassium; TN: Total nitrogen; TP: Total phosphorus; WR: Water repellency cessation time.

* The data of PR and PD were referenced Zhang et al. (2014).

### 2.3 DNA extraction, PCR amplification, and amplicon sequencing

Community DNA was extracted from 0.5 g (fresh weight) soil using the Fast DNA Spin Kit (MP Biomedicals, Carlsbad, CA, USA) following the manufacturer's instructions. The extracted DNA was quantified using a Nanodrop One spectrophotometer (Gene Company Limited, Hong Kong, China).

To compare the soil microbial community composition and diversity in each soil sample, amplicon surveys of a portion of the partial 16S rRNA gene, ITS gene, and *nifH* gene were performed. The barcoded primer sets for 16S rRNA, ITS, and *nifH* gene were 338F/806R (Xu, Tan, & Gai, 2016), ITS1F/2R (Adams, Miletto, & Bruns, 2013), and *nifH*F/*nifH*R (Rösch, Mergel, & Bothe, 2002) targeting the V4‐V5 hypervariable regions of 16S rRNA genes, V1 regions of ITS genes, and a portion of the partial *nifH* genes were used for Illumina sequencing, respectively. Both forward and reverse primers contained Illumina adapters and a 6 bp barcode sequence unique to each sample. Purified DNA (10 ng) from each sample was used as a template for PCR amplification in a 25 μl reaction volume. Thermocycling for 16S rRNA gene was conducted in a Mastercycler pro gradient PCR Cycler (Eppendorf AG, Hamburg, German) as follows: 95°C for 2 min followed by 25 cycles of 94°C for 30 s, 55°C for 30 s, 72°C for 30 s; and a final elongation step at 72°C for 5 min. The PCR amplification of ITS gene was under the following conditions: 95°C for 3 min followed by 30 cycles of 95°C for 30 s, 55°C for 30 s, 72°C for 45 s, ending with a final extension step of 10 min at 72°C. For the *nifH* gene, the PCR conditions were similar with ITS gene except for the degradation temperature was changed to 58°C, and number of cycles was changed to 35. The PCR products were purified using the QIA quick Gel Extraction Kit (Qiagen, Hilden, Germany). Sequencing was conducted on an Illumina Miseq sequencer at Shanghai Majorbio Bio‐pharm Technology Corporation.

### 2.4 Bioinformatic and multivariate statistical analyses

Quality control of the amplicon sequences was performed using QIIME pipeline (version 1.9.0; Caporaso et al., 2010). Briefly, the quality parameters used were as follows: sequences were discarded if they contained any ambiguous base, had more than two mismatches to the primers, one mismatch to the barcode sequence, or a homopolymer longer than 8 bp, minimum sequence length of 150 bp or average quality score of 30. After filtering and chimera removal, de novo operational taxonomic units (OTUs) picking was performed using uclust at 97% sequence identity, and subsequently, taxonomy was assigned to OTU based on the Greengenes database (version 15.13), Unite (version 7.0), Fungene database (RDP Classifier) for 16S rRNA, ITS, *nifH* gene, respectively (Fish et al., 2013; McDonald et al., 2012; Nilsson et al., 2018). Rarefaction was performed with 24,981, 14,050, and 2,722 sequences per sample for the diversity analyses of bacteria, fungi, and diazotrophs. Differences in community structure between samples were visualized using the Bray–Curtis and principal co‐ordinates analysis (PCoA). Distance‐based redundancy analysis (dbRDA), based on Bray–Curtis distances, was used to determine the most significant environmental variables that might influence the microbial community structures using Canoco 5.0 (Microcomputer Power, Ithaca, NY, USA). Before conducted dbRDA analysis, the physicochemical attributes were selected based on the variation inflation factors (VIFs), which stepwise removed redundant attributes, resulting in VIFs of less than 10 (O'brien, 2007). The OTUs detected in at least 10% of the soil samples of a given steppe type were included for downstream analysis. Venn diagrams, constructed by R software, were used to observe the shared and specific OTUs among the different steppe types (Chen & Boutros, 2011). Pearson correlation analysis was performed to evaluate the relationships between environmental factors and microbial community diversity using SPSS (version 13.0).

## 3 RESULTS

### 3.1 Soil properties and plant community

Although the sampling regions spans over 2,000 km, most of the soil samples derived from granite and displayed similar soil pH and similar contents of total and available potassium and phosphorus (TK, TP, AK, AP), while the contents of soil organic matter (SOM), total nitrogen (TN), available nitrogen (AN), and dissolved organic carbon (DOC) were greatly related to the steppe types (Table 1). In comparison, typical and meadow steppe soils displayed significant higher contents of SOM, TN, and AN than desert steppe soils, while no significant differences were detected between typical and meadow steppe soils except for the DOC, which were significantly higher in typical steppe soils than that in meadow soils. The desert steppe soils possessed the highest water repellency, which was about four times higher than the rest steppe soils, while the water repellency of typical steppe soils was slightly lower than that of the meadow soils.

For the plant species, it was detected that *Stipaklemenzii* was the predominant plant species in desert steppe. The dominant plant species in typical steppes were *Leymuschinensis* and *Stipagrandis* while the meadow steppes were dominated by *Leymuschinensis* and *Stipabaicalensis *(Supporting Information Table S1). The average plant coverage (PC) was also significant different among steppe types, with desert steppe displayed the lowest coverage of 19%, followed by typical steppe (average 57%), and the meadow steppe possessed the highest average plant coverage of over 60% (Table 1).

### 3.2 Diversity of soil bacteria, fungi and diazotrophs across steppes

A total of 823,717, 869,800, and 499,108 high‐quality sequences were identified from all soil samples examined before re‐sampling for bacteria, fungi, and *nifH*‐containing bacteria, respectively. These selected sequences were grouped into 2,406, 723, and 453 operational taxonomic units (OTUs) using an arbitrary 97% sequence similarity cutoff after removal of the OTUs occurred in no more than three samples with less than three sequences. All samples were compared at an equivalent sequencing depth of 24,981, 14,050, and 2,722 randomly selected 16S rRNA, ITS and *nifH* gene amplicons per sample. It was shown that the typical and meadow steppes harbored higher OTU numbers and Shannon index of fungi and diazotrophs than desert steppe, indicating that they possessed more diverse fungal and diazotrophic microorganisms than desert steppe, but there were no significant differences in alpha diversity of soil fungal and diazotrophic community between typical and meadow steppe (Table 2). However, for the bacteria, no significant differences in alpha diversity were detected among steppe types.

**Table 2:** Alpha diversity of soil bacterial, fungal, and diazotrophic communities

Steppe type	Bacteria		Fungi		Diazotroph	
	OTU number	Shannon	OTU number	Shannon	OTU number	Shannon
Desert	1632 ± 133^a^	6.14 ± 0.30 a	219 ± 50 b	3.19 ± 0.41 b	112 ± 17 b	3.30 ± 0.34 a
Typical	1722 ± 176 a	6.30 ± 0.08 a	287 ± 69 a	3.62 ± 0.51 a	120 ± 23 ab	3.34 ± 0.14 a
Meadow	1678 ± 49 a	6.32 ± 0.11 a	309 ± 48 a	3.71 ± 0.50 a	142 ± 15 a	3.35 ± 0.29 a

Notes

Different letters (a, b) in the same column represent significant differences at *p* < 0.05.

a Values represent *M* ± *SD* (*n* = 5).

### 3.3 Distribution patterns of soil bacteria, fungi, and diazotrophs across steppes

**Figure 1:**
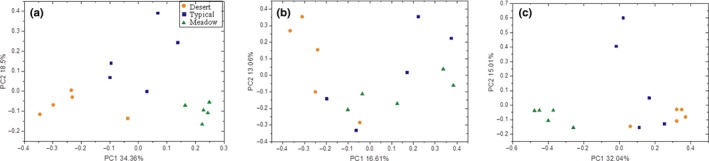
Principal coordinate analysis with Bray–Curtis dissimilarity of the community structures of soil bacteria (a), fungi (b), and diazotrophs (c) across steppes

The community structures of soil bacteria, fungi, and *nifH*‐containing bacteria across different steppe types were evaluated using principal co‐ordinates analysis (PCoA). The results showed that the communities of soil bacteria, fungi, and diazotrophs obviously structured according to the types of steppes, but these three microbial community groups varied slightly (Figure 1). Both bacterial and diazotrophic communities were clearly clustered based on the steppe types, while fungi exhibited a different feature that only desert steppe was clearly departed from typical and meadow steppes that were mixed without clear separations. ANOSIM analysis also showed that there were significant differences in the community structures of bacteria (*r *= 0.6809, *p* = 0.001) and diazotrophs (*r* = 0.6613, *p* = 0.001). But for the fungi, significant differences in community structures were detected between desert and typical (*r* = 0.32, *p* = 0.031) or meadow steppe soils (*r* = 0.552, *p* = 0.024), while no significant differences were observed between typical and meadow steppe soils (*r* = 0.2, *p* = 0.094).

**Figure 2:**
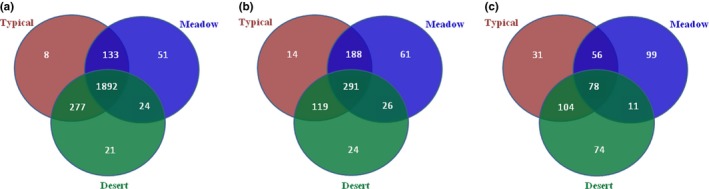
Venn plots indicating the unique and overlap operational taxonomic units in soil bacteria (a), fungi (b), and diazotrophs (c) across steppes

In order to further explore whether the differences in microbial community structures were caused by the changes in the community compositions or the shifts in the assembling patterns of each microbial component (relative abundance), we split the overall community into three general categories; “common”—OTUs detected in all types of steppe soils, “unique”—OTUs found only in one specific type of steppe soils, and “bi‐shared”—OTUs shared by two types of steppe soils (Figure 2). Among them, the proportion of common OTUs to total OTU numbers of bacteria, fungi, and diazotrophs in three types of steppes were 78.64%, 40.25%, and 17.22%, respectively, suggesting that bacteria were significantly more homologous than fungi among the steppes, and the functional *nifH*‐containing communities showed the highest heterogeneity.

It was further detected that bacteria, fungi, and diazotrophs presented differential adaptations to the three types of steppes (Figure 2a, Supporting Information Table S2). Most of bacterial populations were the common communities, the ratios of common groups to the total community of bacteria in desert, typical, and meadow steppes were 85.46%, 81.90%, and 90.10%, respectively, indicating that the bacterial communities were quite evenly distributed among these grassland soils. The unique bacterial OTUs in each of the steppes were ignorable, with less than 3% proportion of the whole bacterial community in each steppe. The rest OTUs were shared either by desert and typical steppes (277 OTUs, average 12.25%) or by typical and meadow steppes (133 OTUs, average 6.05%), but only 24 OTUs were shared only by desert and meadow steppes.

However, fungi communities presented an obviously different picture, the ratios of common groups to the total OTUs in desert, typical, and meadow steppes were 63.26%, 47.55%, and 51.41%, respectively. The proportions of unique fungal OTUs to total fungal community were obviously higher than bacteria, but varied significantly depends on steppe types, which were 5.22%, 2.29%, and 10.78% in desert, typical, and meadow steppe, correspondingly. Similarly, merely 26 fungal OTUs were shared only by desert and meadow steppes, the highest bi‐shared fungal groups happened between typical and meadow steppes, which took about average 30.72% and 33.22% of fungal community numbers in typical and meadow steppe, respectively (Figure 2b, Supporting Information Table S2).

For the functional diazotrophs, it was detected that the proportion of common *nifH*‐containing community to the whole community was significantly lower than bacteria and fungi communities. The proportions of common to total OTUs in desert, typical, and meadow steppes were only 29.21%, 29.00%, and 31.97%, respectively. The unique N_2_ fixers increased to dominant position in each steppe, which took 27.72%, 11.52%, and 40.57% of total *nifH* OTUs in desert, typical, and meadow steppe, respectively. Likewise, the only 11 OTUs were bi‐shared by desert and meadow steppes. Most of *nifH* OTUs were shared either by desert and typical steppes or by typical and meadow steppes; the bi‐shared communities took about 38.95%, 59.48%, and 22.95% of *nifH*‐containing bacteria in desert, typical, and meadow steppes, correspondingly (Figure 2c, Supporting Information Table S2).

**Figure 3:**
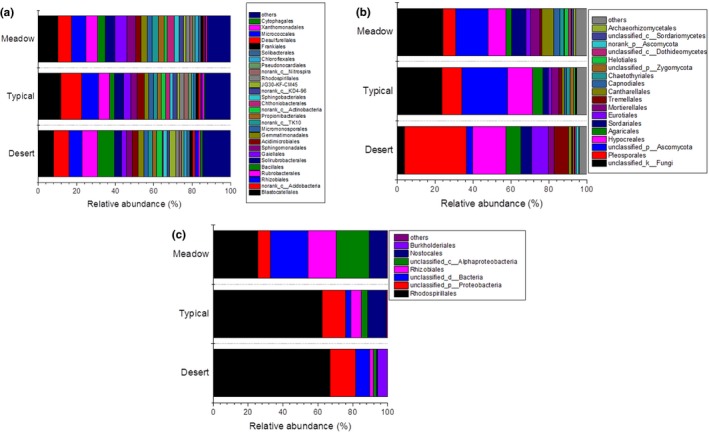
Relative abundance of (a) soil bacterial, (b) fungal, and (c) diazotrophic communities on order level. Vertical columns represent soils from different steppes; horizontal rows represent orders that relative abundance >1% in at least one steppe habitat for bacteria and fungi, and all the orders of diazotrophic communities were presented

Taxonomy analysis was further conducted to display the compositions and structures of bacteria, fungi, and diazotrophs across steppes. The results showed that different type of steppe soils shared similar bacterial orders (Figure 3a). Bacterial orders of *Blastocatellales*, *Rhizobiales*, *Rubrobacterales*, and unclassified *Acidobacteria* were dominant groups (average relative abundance >5%) across all steppe types; they evenly distributed in three types of steppes without significant differences. However, there were also some less abundant (relative abundance <5%) orders differentially distributed in different steppe types. For example, *Chthoniobacterales* and *Gaiellales* were more abundant in meadow soils, followed by typical steppe soils, and the desert steppe soils displayed the lowest proportion. Some groups such as *Bacillales *and *JG30‐KF‐CM45*, showed higher relative abundance in desert steppe than that in other two steppes. Besides, it was worth noting that, among the taxa that significantly differentially distributed among steppes, there were 18 out of 24 bacterial orders were rare taxa with relative abundance less than 0.5% (Supporting Information Figure S2a), indicating that the rare taxa would respond more to the habitat changes than the dominant bacterial groups. But for the fungal community, it was detected that the fungal composition in desert steppe was significantly differed from other two steppe soils while the fungal community structures of typical and meadow steppes were relatively similar (Figure 3b). The most outstanding features were that the dominant orders of *Pleosporales* and *Tremellales *in desert steppe soils were significantly reduced in typical and meadow steppe soils. On the contrary, the average relative abundance of unclassified order that affiliated to *Ascomycota* phylum increased from 4.39% in desert steppe to 21.94% and 17.06% in typical and meadow steppe soils, respectively. Besides, the relative abundance of some less abundant orders such as *Cantharellales*, *Capnodiales*, and *Chaetothyriales* were also significantly higher in typical and meadow steppe soils than that in desert steppe, especially the rare taxa with relative abundance less than 0.5%, varied more than the dominant groups (Supporting Information Figure S2b). The community composition structures of functional diazotrophs exhibited a different pattern (Figure 3c). Although most of the dominant diazotrophic groups were similar among the three steppes, the proportions of them varied between the steppe types, such as *Rhodosprillales* was an overwhelming dominant component in desert steppe with average proportion of 67.01%, but its relative abundance reduced obviously in the other steppes, especially in meadow steppe (average 25.46%). The *Burkholderiales* was detected in desert steppe soils with average proportion of 5.72%, which was nearly undetected in other two steppes. Besides, the groups of unclassified *Alphaproteobacteria* and *Nostocales* were minority groups in desert steppe but were dominant in both typical and meadow steppes. For the taxa with relative abundance less than 0.5%, there were 3 out of 4 *nifH*‐containing bacterial orders only detected in one or two specific steppe soils (Supporting Information Figure S2c), suggesting that most of the rare diazotrophic communities required specific habitat environment.

### 3.4 Explaining variance in soil bacterial, fungal, and diazotrophic communities

**Figure 4:**
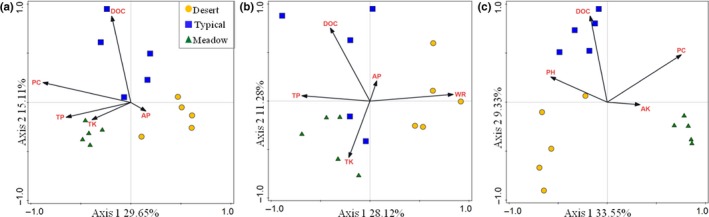
Distance‐based RDA analysis showing the relationships between environmental factors and community structures of soil bacteria (a), fungi (b), and diazotrophs (c) across steppes

DbRDA analysis was used to further analyze how individual environmental factors influenced the relative abundances of bacterial, fungal, and diazotrophic communities. Results revealed that the examined environmental factors could explain 75.4%, 65.2%, and 70.1% of the variations in the community structures of bacteria, fungi, and diazotrophs across steppes, respectively (Table 3). Among them, PC, DOC, and TP were significant factors in shaping bacterial community structure (Figure 4a); these three factors could explain 46.2% of the total variation (Table 3). The community structure of fungi was significantly correlated with WR, DOC, and TP; these parameters could explain 34.7% of the fungal community variation (Figure 4b). But for the diazotrophic communities, it was predominantly shaped by PC, followed by DOC and AK, which could explain 21.9%, 10.4%, and 9.8% of the total variation in diazotrophic communities (Figure 4c, Table 3).

**Table 3:** Contributions of environmental factors to explain the variations in microbial community structures across steppes

	Bacteria		Fungi		Diazotrophs	
	Explains %^a^	*p*‐Value	Explains %	*p*‐Value	Explains %	*p*‐Value
PC	24.3^b^	0.002	5.4	0.538	21.9^c^	0.012
DOC	11.8^c^	0.014	10.5^c^	0.014	10.4	0.102
TP	10.1^c^	0.016	10.1^c^	0.024	6.4	0.234
TK	7.4	0.09	6.6	0.298	6.1	0.268
AP	6.6	0.142	7.1	0.196	4.8	0.422
WR	6.2	0.15	14.1^b^	0.004	3.2	0.704
AK	4.1	0.44	5.4	0.522	9.8	0.08
PH	3.9	0.544	6	0.44	7.5	0.172
Total	74.4		65.2		70.1	

Notes

Environmental factors: AK: Available potassium; AP: Available phosphorus; DOC: dissolved organic carbon; PC: Plant coverage; TK: Total potassium; TP: Total phosphorus; WR: Water repellency cessation time.

a The proportion of total variations in microbial communities explained by each environmental factor. Forward selection on 499 permutations was used to test the significant contributions of each factor.

b Represent significant at 0.01 level.

c Indicates significant at 0.05 level.

Pearson correlation analysis was performed to evaluate the relationships between environmental factors and microbial community diversity (Supporting Information Table S3). The results showed that there were no significant correlations between soil bacterial diversity and any soil or climate factors. However, significantly positive correlations were detected between fungal diversity and MAP (*r* = 0.586, *p* < 0.05), PR (*r* = 0.542, *p* < 0.05) and PD (*r* = 0.519, *p* < 0.05), while the PR (*r* = 0.592, *p* < 0.05) and PD (*r* = 0.595, *p* < 0.05) showed significantly positive correlation with the diversity of diazotrophs.

## 4 DISCUSSION

In this study, the soil samples were randomly selected in the grasslands covering desert, typical, and meadow steppes in Inner Mongolia. The formation of different steppes is largely depended on MAT and MAP (Yan et al., 2015). Since each type of grasslands have been developed hundreds of years and formed unique characteristics, the biomass and species richness of plant community were significantly different between the steppes (Yan et al., 2015; Zhang et al., 2014). These heterogeneous environments would induce various resident microorganisms among them.

This work was focused on the distributions of the communities of bacteria, fungi, and diazotrophs in natural steppes. We found that different microbial groups exhibited various distribution patterns across the grasslands. Firstly, most of bacterial species were homogeneously shared by the desert, typical, and meadow steppes and the unique species in specific steppe were very few. Although it has been reported that soil characteristics were important factors in shaping soil bacterial communities (Griffiths et al., 2011; Lauber et al., 2009), climate conditions and plantation could also cause some shifts of bacterial communities (Leff et al., 2018; Wang et al., 2015), interestingly, these factors did not show clear influence on the bacterial compositions in the steppes in the current work. The question is which factor would be more important in relation to the distributions of bacterial compositions between the steppes. Among the variables of climate, plant traits, soil nutrient contents, grazing density, and soil types, it was found that almost all the soil samples shared the same parent material background of granite. It was manifested by similar soil pH, TP, and TK contents (Table 1), which are closely related to the nature of parent materials (Blanchet et al., 2017; Imaya, Ohta, Tanaka, & Inagaki, 2005; Kooijman, Jongejans, & Sevink, 2005). Previous studies have suggested that soil parent material played significant roles in determining bacterial community composition via controlling soil characteristics (Sun et al., 2015; Ulrich & Becker, 2006), soils derived from same parent material displayed similar community composition even they distributed thousands of kilometers away (Sheng et al., 2015). Therefore, we speculated that soil parent material‐derived properties might be important factors in shaping bacterial compositions in various steppes. However, despite the bacterial compositions were homogenous among steppes, some clear changes in the relative abundances of some bacterial groups existed among steppes, resulted in significant changes in their community structures. Meanwhile, the changes in plant traits and soil nutrient conditions were suggested to be the predominant factors in regulating the relative abundance (Figure 4a). This was in agreement with previous studies showing that the changes in habitat environments (climate, soil fertility, aboveground plant traits) could influence the growth of bacterial communities (Hu et al., 2014; Leff et al., 2018; Singh et al., 2007; Wang et al., 2015).

Previous study suggested that fungal community distribution was primarily driven by precipitation in Inner Mongolia temperate grasslands (Wang et al., 2018). Although we know little about whether the climate condition exerts direct effects on fungal community compositions, it was clear that the climate conditions played major roles in the formation of different steppes in Inner Mongolia (Yan et al., 2015). Since fungal communities are largely relied on soil organic matters and the plant diversity (Chen et al.,; Zak, Holmes, & Tilman, 2003), their compositions might be closely related to the types of steppes, as different plant communities offer different amount and heterogeneity of resources to fungal communities (Bardgett et al., 2005; Duffy et al., 2007). Our results showed that the fungal community compositions and structures in typical and meadow steppes were similar, but which were significantly different from that in desert steppe. It was also found that the typical and meadow steppes possessed similar soil carbon and nitrogen contents, which were significantly higher than that in desert steppe. These soil characteristics were mainly derived from the residues of aboveground plants in natural steppe ecosystems. Besides, it was also discovered that typical and meadow steppes possessed more similarities in plant traits when compared to desert steppe, including plant coverage and plant species. Coincidently, the dbRDA analysis also showed the DOC content was a significant factor correlated with the soil fungal community structure. Therefore, accumulation effects of plant residues and root excretions might be an important determining factor for the fungal community compositions and structures across the steppes. It was worth noting that we also detected a significant correlation between water repellency (WR) and fungal community structure (Figure 4b, Table 3). Previous studies have documented that WR plays important roles in water retention and water conductivity in arid and semi‐arid regions (Yang et al., 2014). There are many factors, such as climate, plant traits, soil characteristics, and microorganisms, can affect WR development (Atanassova & Doerr, 2011; Lozano et al., 2013; Rillig, Noorf, & Pedrom, 2010). On the other side, the formed soil WR will subsequently regulate soil fungal distribution via influencing soil water holding capacity, soil fertility, and plant growth (Goebel, Bachmann, & Guggenberger, 2011). Therefore, WR could also be a suitable indicator for predicting the variations in the soil microbial communities in the steppes (Braun, Böckelmann, & Szewzyk, 2011).

However, although *nifH*‐containing microorganisms are also bacteria, their distribution was largely relied on the steppe types, the species were either unique or bi‐shared in the steppes and the common group took a very limited proportion. This result suggested that the distribution pattern of this functional group might be driven by different mechanisms compared to the whole bacterial community. Such a scenario might be due to that its population size is relatively smaller when compared to the whole bacterial community, they may respond more sensitively to the changes of habitat environment (Henry et al., 2006; Stone et al., 2015). It has been indicated that the community structure and function of diazotrophs were strongly linked to soil quality and plant species (Wang, Li, Li, & Li, 2017; Zhang, Li, Wang, Xiao, & Liu, 2006). High plant species richness and biomass and high soil fertility could induce high diversity of *nifH*‐containing communities (Köberl et al., 2016; Tu et al., 2016). In this study, it was found that the diversity of *nifH*‐containing communities was significantly correlated with plant diversity and richness, with the meadow steppe possessing the highest plant species richness and plant biomass, followed by typical and desert steppe. The soil microbial habitat environment influenced by different aboveground plant communities may selectively promote the growth of diazotrophic communities (Hamelin, Fromin, & Aragno, 2002). Besides, the dbRDA analysis also showed the diazotrophic community structure was predominantly shaped by plant traits and DOC content. Therefore, soil fertility and plant species components would be major driving factors in regulating the compositions and structures of *nifH*‐containing microbial communities.

It was also observed that the proportion of bi‐shared OTUs of bacteria, fungi, and diazotrophs between desert and meadow steppes were minimum, but the proportions either between desert and typical steppes or between typical and meadow steppes were significantly high. Although we have little knowledge about these phenomena, there should be some transient properties between these ecosystems affected the distributions of microbial communities (Yao et al., 2017). The geographic locations of the steppes follow the order of meadow, typical, and desert steppes from the east to the west of Inner Mongolia. This would imply a possible interpretation, the desert and typical steppes, and typical and meadow steppes were adjacent; they possessed high bi‐shared microbial communities (Wang et al., 2015). Furthermore, few bi‐shared microbial communities that detected between these two ecosystems might also be linked to the transitions of a few grass species and climate conditions between them (Yan et al., 2015).

## 5 CONCLUSIONS

The community compositions of soil bacteria, fungi, and diazotrophs exhibited different responses to the habitat changes caused by steppe types. The community compositions of bacteria were homologous across steppes, which might be explained by the similar soil parent material background. But the habitat heterogeneity induced shifts in the relative abundance of some bacterial groups, which resulted in significant differences in community structures among steppes. Typical and meadow steppe soils possessed similar fungal community structure, which were significantly different from desert steppe soils, and the soil nutrient status, water repellency, and plant traits would be the key determinants in shaping soil fungal communities in the steppe ecosystems. The community compositions and structures of diazotrophs were largely depended on steppe types, which could be mainly explained by the variation in aboveground plant traits and soil fertility across the steppes.

## CONFLICT OF INTEREST

None declared.

## AUTHOR CONTRIBUTIONS

R.S. designed and performed the study, analyzed data, and wrote the manuscript; K.L. W.Z.Z., H.X.W., and X.Q.Z. performed parts of the experiment; H.W., H.L.L, X.Y.Z., Q.M.L., X.C.S., Y.F.T, L.A. W.X.W., and L.A. participated in the study design and sample collection; W.X.W. and L.A. edited the manuscript. All authors read and approved the final manuscript.

## Data Availability

The dataset supporting the conclusions of this article is available at NCBI SRA, https://www.ncbi.nlm.nih.gov/sra/PRJNA498380.

## References

[ece34940-bib-0001] Adams, R. I. , Miletto, M. , Taylor, J. W. , & Bruns, T. D. (2013). Dispersal in microbes: Fungi in indoor air are dominated by outdoor air and show dispersal limitation at short distances. The ISME Journal, 7(7), 1262–1273. 10.1038/ismej.2013.28 23426013 PMC3695294

[ece34940-bib-0002] Atanassova, I. , & Doerr, S. H. (2011). Changes in soil organic compound composition associated with heat‐induced increases in soil water repellency. European Journal of Soil Science, 62, 516–532. 10.1111/j.1365-2389.2011.01350.x

[ece34940-bib-0003] Bao, S. D. (2000). Analysis of soil characteristics. Beijing, China: Chinese Agricultural Press.

[ece34940-bib-0004] Bardgett, R. D. , Bowman, W. D. , Kaufmann, R. , & Schmidt, S. K. (2005). A temporal approach to linking aboveground and belowground ecology. Trends in Ecology and Evolution, 20(11), 634–641. 10.1016/j.tree.2005.08.005 16701447

[ece34940-bib-0005] Blanchet, G. , Libohova, Z. , Joost, S. , Rossier, N. , Schneider, A. , Jeangros, B. , & Sinaj, S. (2017). Spatial variability of potassium in agricultural soils of the canton of Fribourg, Switzerland. Geoderma, 290, 107–121. 10.1016/j.geoderma.2016.12.002

[ece34940-bib-0006] Braun, B. , Böckelmann, U. , Grohmann, E. , & Szewzyk, U. (2011). Bacterial soil communities affected by water‐repellency. Geoderma, 158, 343–351. 10.1016/j.geoderma.2010.06.001

[ece34940-bib-0007] Caporaso, J. G. , Kuczynski, J. , Stombaugh, J. , Bittinger, K. , Bushman, F. D. , Costello, E. K. , & Knight, R. (2010). QIIME allows analysis of high‐throughput community sequencing data. Nature Methods, 7(5), 335–336. 10.1038/nmeth.f.303 20383131 PMC3156573

[ece34940-bib-0008] Chen, H. , & Boutros, P. C. (2011). Venn Diagram: A package for the generation of highly‐customizable Venn and Euler diagrams in R. BMC Bioinformatics, 12(1), 35 10.1186/1471-2105-12-35 21269502 PMC3041657

[ece34940-bib-0009] Chen, Y. L. , Ding, J. Z. , Peng, Y. F. , Li, F. , Yang, G. B. , Qin, S. Q. , … Yang, Y. H. (2016). Patterns and drivers of soil microbial communities in Tibetan alpine and global terrestrial ecosystems. Journal of Biogeography, 43(10), 2027–2039. 10.1111/jbi.12806

[ece34940-bib-0010] Dart, P. J. , & Wani, S. P. (1982). IN *Non-symbiotic nitrogen fixation and soil fertility*. In Non-Symbiotic nitrogen fixation and organic matter in the tropics. Symposium Papers I, Transactions, 12th International Congress on Soil Science, New Delhi, pp. 3–27.

[ece34940-bib-0011] Degrune, F. , Theodorakopoulos, N. , Colinet, G. , Hiel, M. P. , Bodson, B. , Taminiau, B. , … Hartmann, M. (2017). Temporal dynamics of soil microbial communities below the seedbed under two contrasting tillage regimes. Frontiers in Microbiology, 8, 1–15. 10.3389/fmicb.2017.01127 28674527 PMC5474472

[ece34940-bib-0012] Duffy, J. E. , Cardinale, B. J. , France, K. E. , McIntyre, P. B. , Thebault, E. , & Loreau, M. (2007). The functional role of biodiversity in ecosystems: Incorporating trophic complexity. Ecology Letters, 10, 522–538. 10.1111/j.1461-0248.2007.01037.x 17498151

[ece34940-bib-0013] Fierer, N. , & Ladau, J. (2012). Predicting microbial distributions in space and time. Nature Methods, 9(6), 549 10.1038/nmeth.2041 22669651

[ece34940-bib-0014] Filion, M. , St-Arnaud, M. , & Fortin, J. A. (1999). Direct interaction between the arbuscular mycorrhizal fungus Glomus intraradices and different rhizosphere microorganisms. New Phytologist, 141, 525–533. 10.1046/j.1469-8137.1999.00366.x

[ece34940-bib-0015] Fish, J. A. , Chai, B. , Wang, Q. , Sun, Y. , Brown, C. T. , Tiedje, J. M. , & Cole, J. R. (2013). FunGene: The functional gene pipeline and repository. Frontiers in Microbiology, 4, 291 10.3389/fmicb.2013.00291 24101916 PMC3787254

[ece34940-bib-0016] Goebel, M. O. , Bachmann, J. , Reichstein, M. , Janssens, I. A. , & Guggenberger, G. (2011). Soil water repellency and its implications for organic matter decomposition—Is there a link to extreme climatic events? Global Change Biology, 17, 2640–2656. 10.1111/j.1365-2486.2011.02414.x

[ece34940-bib-0017] Griffiths, R. I. , Thomson, B. C. , James, P. , Bell, T. , Bailey, M. , & Whiteley, A. S. (2011). The bacterial biogeography of British soils. Environmental Microbiology, 13, 1642–1654. 10.1111/j.1462-2920.2011.02480.x 21507180

[ece34940-bib-0018] Hamelin, J. , Fromin, N. , Tarnawski, S. , Teyssier‐Cuvelle, S. , & Aragno, M. (2002). nifH gene diversity in the bacterial community associated with the rhizosphere of *Molinia coerulea*, an oligonitrophilic perennial grass. Environmental Microbiology, 4(8), 477–481. 10.1046/j.1462-2920.2002.00319.x 12153588

[ece34940-bib-0019] Hanson, C. A. , Fuhrman, J. A. , Horner‐Devine, M. C. , & Martiny, J. B. (2012). Beyond biogeographic patterns: Processes shaping the microbial landscape. Nature Reviews Microbiology, 10, 497–506. 10.1038/nrmicro2795 22580365

[ece34940-bib-0020] Hazard, C. , Gosling, P. , van der Gast, C. J. , Mitchell, D. T. , Doohan, F. M. , & Bending, G. D. (2013). The role of local environment and geographical distance in determining community composition of arbuscular mycorrhizal fungi at the landscape scale. The ISME Journal, 7, 498–508. 10.1038/ismej.2012.127 23096401 PMC3578569

[ece34940-bib-0021] Henry, S. , Bru, D. , Stres, B. , Hallet, S. , & Philippot, L. (2006). Quantitative detection of the nosZ gene, encoding nitrous oxide reductase, and comparison of the abundances of 16S rRNA, *narG*, *nirK*, and *nosZ* genes in soils. Applied Environmental Microbiology, 72, 5181–5189. 10.1128/AEM.00231-06 16885263 PMC1538733

[ece34940-bib-0022] Hu, Y. , Xiang, D. , Veresoglou, S. D. , Chen, F. , Chen, Y. , Hao, Z. , … Chen, B. (2014). Soil organic carbon and soil structure are driving microbial abundance and community composition across the arid and semi‐arid grasslands in northern China. Soil Biology and Biochemistry, 77, 51–57. 10.1016/j.soilbio.2014.06.014

[ece34940-bib-0023] Imaya, A. , Ohta, S. , Tanaka, T. , & Inagaki, Y. (2005). General chemical properties of brown forest soils developed from different parent materials in the submontane zone of the Kanto and Chubu districts, Japan. Soil Science and Plant Nutrition, 51, 873–884. 10.1111/j.1747-0765.2005.tb00122.x

[ece34940-bib-0024] Kalembas, S. J. , & Jenkinso, D. S. (1973). Comparative study of titrimetric and gravimetric methods for determination of organic carbon in soil. Journal of the Science of Food and Agriculture, 24, 1085–1090. 10.1002/jsfa.2740240910

[ece34940-bib-0025] Kang, L. , Han, X. G. , Zhang, Z. B. , & Sun, O. J. (2007). Grassland ecosystems in China: Review of current knowledge and research advancement. Philosophical Transactions of the Royal Society B: Biological Sciences, 362, 997–1008. 10.1098/rstb.2007.2029 PMC2435566 17317645

[ece34940-bib-0026] Kennedy, I. R. , & Islam, N. (2001). The current and potential contribution of asymbiotic nitrogen fixation to nitrogen requirements on farms: A review. Australian Journal of Experimental Agriculture, 41(3), 447–457. 10.1071/EA00081

[ece34940-bib-0027] King, A. J. , Farrer, E. C. , & Schmidt, S. K. (2012). Co‐occurrence patterns of plants and soil bacteria in the high‐alpine subnival zone track environmental harshness. Frontiers in Microbiology, 3, 347 10.3389/fmicb.2012.00347 23087675 PMC3469205

[ece34940-bib-0028] King, A. J. , Freeman, K. R. , McCormick, K. F. , Lynch, R. C. , Lozupone, C. , Knight, R. , & Schmidt, S. K. (2010). Biogeography and habitat modelling of high‐alpine bacteria. Nature Communications, 1, 53 10.1038/ncomms1055 20975720

[ece34940-bib-0029] Klein, M. , Swinnen, S. , Thevelein, J. M. , & Nevoiqt, E. (2017). Glycerol metabolism and transport in yeast and fungi: Established knowledge and ambiguities. Environmental Microbiology, 19(3), 878–893. 10.1111/1462-2920.13617 27878932

[ece34940-bib-0030] Köberl, M. , Erlacher, A. , Ramadan, E. M. , El‐Arabi, T. F. , Müller, H. , Bragina, A. , & Berg, G. (2016). Comparisons of diazotrophic communities in native and agricultural desert ecosystems reveal plants as important drivers in diversity. FEMS Microbiology Ecology, 92, fiv166 10.1093/femsec/fiv166 26705571 PMC4730177

[ece34940-bib-0031] Kooijman, A. M. , Jongejans, J. , & Sevink, J. (2005). Parent material effects on Mediterranean woodland ecosystem in NE Spain. Catena, 59, 55–68. 10.1016/j.catena.2004.05.004

[ece34940-bib-0032] Lauber, C. L. , Hamady, M. , Knight, R. , & Fierer, N. (2009). Pyrosequencing based assessment of soil pH as a predictor of soil bacterial community structure at the continental scale. Applied Environmental Microbiology, 75, 5111–5120. 10.1128/AEM.00335-09 19502440 PMC2725504

[ece34940-bib-0033] Leff, J. W. , Bardgett, R. D. , Wikinson, A. , Jackson, B. G. , Pritchard, W. J. , De Long, J. R. , & Fierer, N. (2018). Predicting the structure of soil communities from plant community taxonomy, phylogeny, and traits. The ISME Journal, 12(7), 1794–1805. 10.1038/s41396-018-0089-x 29523892 PMC6004312

[ece34940-bib-0034] Lozano, E. , Jiménez‐Pinillaa, P. , Mataix‐Soleraa, J. , Arceneguia, V. , Bárcenasb, G. M. , González‐Pérezc, J. A. , … Mataix‐Beneyto, J. (2013). Biological and chemical factors controlling the patchy distribution of soil water repellency among plant species in a Mediterranean semiarid forest. Geoderma, 207–208(5), 212–220. 10.1016/j.geoderma.2013.05.021

[ece34940-bib-0035] Lozupone, C. A. , & Knight, R. (2007). Global patterns in bacterial diversity. Proceedings of the National Academy of Sciences of the United States of America, 104(27), 11436–11440. 10.1073/pnas.0611525104 17592124 PMC2040916

[ece34940-bib-0036] McDonald, D. , Price, M. N. , Goodrich, J. , Nawrocki, E. P. , DeSantis, T. Z. , Probst, A. , … Hugenholtz, P. (2012). An improved Greengenes taxonomy with explicit ranks for ecological and evolutionary analyses of bacteria and archaea. The ISME Journal, 6(3), 610–618. 10.1038/ismej.2011.139 22134646 PMC3280142

[ece34940-bib-0037] Ni, J. , & Zhang, X. S. (2000). Climate variability, ecological gradient and the Northeast China Transect (NECT). Journal of Arid Environment, 46, 313–325. 10.1006/jare.2000.0667

[ece34940-bib-0038] Nilsson, R. H. , Larsson, K.‐H. , Taylor, A. F. S. , Bengtsson‐Palme, J. , Jeppesen, T. S. , Schigel, D. , … Abarenkov, K. (2018). The UNITE database for molecular identification of fungi: Handling dark taxa and parallel taxonomic classifications. Nucleic Acids Research, 47(D1), D259–D264. 10.1093/nar/gky1022 PMC6324048 30371820

[ece34940-bib-0039] Pellissier, L. , Niculita‐Hirzel, H. , Dubuis, A. , Pagni, M. , Guex, N. , Ndiribe, C. , … Guisan, A. (2014). Soil fungal communities of grasslands are environmentally structured at a regional scale in the Alps. Molecular Ecology, 23, 4274–4290. 10.1111/mec.12854 25041483

[ece34940-bib-0040] Prober, S. M. , Leff, J. W. , Bates, S. T. , Borer, E. T. , Firn, J. , Harpole, W. S. , … Fierer, N. (2015). Plant diversity predicts beta but not alpha diversity of soil microbes across grasslands worldwide. Ecology Letters, 18, 85–95. 10.1111/ele.12381 25430889

[ece34940-bib-0041] Rillig, M. C. , Noorf, M. , Evaf, L. , & Pedrom, A. (2010). Mycelium of arbuscular mycorrhizal fungi increases soil water repellency and is sufficient to maintain water‐stable soil aggregates. Soil Biology and Biochemistry, 42, 1189–1191. 10.1016/j.soilbio.2010.03.027

[ece34940-bib-0042] O'brien, R. M. (2007). A caution regarding rules of thumb for variance inflation factors. Quality and Quantity, 41(5), 673–690. 10.1007/s11135-006-9018-6

[ece34940-bib-0043] Rösch, C. , Mergel, A. , & Bothe, H. (2002). Biodiversity of denitrifying and dinitrogen‐fixing bacteria in an acid forest soil. Applied and Environmental Microbiology, 68(8), 3818–3829. 10.1128/AEM.68.8.3818-3829.2002 12147477 PMC124007

[ece34940-bib-0044] Rousk, J. , Brookes, P. C. , & Bååth, E. (2009). Contrasting soil pH effects on fungal and bacterial growth suggest functional redundancy in carbon mineralization. Applied Environmental Microbiology, 75(6), 1589–1596. 10.1128/AEM.02775-08 19151179 PMC2655475

[ece34940-bib-0045] Rudnick, M. B. , van Veen, J. A. , & de Boer, W. (2015). Baiting of rhizosphere bacteria with hyphae of common soil fungi reveals a diverse group of potentially mycophagous secondary consumer. Soil Biology and Biochemistry, 177, 859–876. 10.1016/j.soilbio.2015.04.015

[ece34940-bib-0046] Sheng, R. , Meng, D. L. , Wu, M. N. , Di, H. J. , Qin, H. L. , & Wei, W. X. (2013). Effect of agricultural land use change on community composition of bacteria and ammonia oxidizers. Journal of Soils and Sediments, 13, 1246–1256. 10.1007/s11368-013-0713-3

[ece34940-bib-0047] Sheng, R. , Qin, H. L. , O'Donnell, A. G. , Huang, S. , Wu, J. S. , & Wei, W. X. (2015). Bacterial succession in paddy soils derived from different parent materials. Journal of Soils and Sediments, 15(4), 982–992. 10.1007/s11368-014-1058-2

[ece34940-bib-0048] Singh, B. K. , Munro, S. , Potts, J. M. , & Millard, P. (2007). Influence of grass species and soil type on rhizosphere microbial community structure in grassland soils. Applied Soil Ecology, 36, 147–155. 10.1016/j.apsoil.2007.01.004

[ece34940-bib-0049] Stone, M. M. , Kan, J. , & Plante, A. F. (2015). Parent material and vegetation influence bacterial community structure and nitrogen functional genes along deep tropical soil profiles at the Luquillo Critical Zone Observatory. Soil Biology and Biochemistry, 80, 273–282. 10.1016/j.soilbio.2014.10.019

[ece34940-bib-0050] Strickland, M. S. , & Rousk, J. (2010). Considering fungal:bacterial dominance in soils—Methods, controls, and ecosystem implications. Soil Biology and Biochemistry, 42, 1385–1395. 10.1016/j.soilbio.2010.05.007

[ece34940-bib-0051] Sun, L. , Gao, J. S. , Huang, T. , Kendall, J. R. A. , Shen, Q. R. , & Zhang, R. F. (2015). Parent material and cultivation determine soil bacterial community structure and fertility. FEMS Microbiololgy Ecology, 91(1), 1–10. 10.1093/femsec/fiu010 25764534

[ece34940-bib-0052] Tedersoo, L. , & Bahram, M. (2016). Tree diversity and species identity effects on soil fungi, protists and animals are context dependent. The ISME Journal, 10, 346e362 10.1038/ismej.2015.116 26172210 PMC4737927

[ece34940-bib-0054] Tillman, R. W. , Scotter, D. R. , Wallis, M. G. , & Clothier, B. E. (1989). Water‐repellency and its measurement by using intrinsic sorptivity. Australian Journal of Soil Research, 27, 637–644. 10.1071/SR9890637

[ece34940-bib-0055] Tu, Q. , Deng, Y. , Yan, Q. , Shen, L. , He, Z. , Wu, L. , … Zhou, J. (2016). Biogeographic patterns of soil diazotrophic communities across six forests in the North America. Molecular Ecology, 25, 2937–2948. 10.1111/mec.13651 27085668

[ece34940-bib-0056] Ulrich, A. , & Becker, R. (2006). Soil parent material is a key determinant of the bacterial community structure in arable soils. FEMS Microbiology Ecology, 56, 430–443. 10.1111/j.1574-6941.2006.00085.x 16689875

[ece34940-bib-0057] Van Der Heijden, M. , Bardgett, R. , & van Straalen, N. (2008). The unseen majority: Soil microbes as drivers of plant diversity and productivity in terrestrial ecosystems. Ecology Letters, 11, 296–310. 10.1111/j.1461-0248.2007.01139.x 18047587

[ece34940-bib-0058] Wang, D. , Rui, Y. , Ding, K. , Cui, X. , Hao, Y. , Tang, L. , … Wang, Y. (2018). Precipitation drives the biogeographic distribution of soil fungal community in Inner Mongolian temperate grasslands. Journal of Soils and Sediments, 18, 222–228. 10.1007/s11368-017-1727-z

[ece34940-bib-0059] Wang, X. , Van Nostrand, J. D. , Deng, Y. , Lü, X. T. , Wang, C. , Zhou, J. , & Han, X. G. (2015). Scale‐dependent effects of climate and geographic distance on bacterial diversity patterns across northern China's grasslands. FEMS Microbiology Ecology, 91(12), 1–10. 10.1093/femsec/fiv133 26519142

[ece34940-bib-0060] Wang, Y. , Li, H. , Li, J. , & Li, X. (2017). The diversity and co‐occurrence patterns of diazotrophs in the steppes of Inner Mongolia. Catena, 157, 130–138. 10.1016/j.catena.2017.05.006

[ece34940-bib-0061] Xu, N. , Tan, G. , Wang, H. , & Gai, X. (2016). Effect of biochar additions to soil on nitrogen leaching, microbial biomass and bacterial community structure. European Journal of Soil Biology, 74, 1–8. 10.1016/j.ejsobi.2016.02.004

[ece34940-bib-0062] Yan, H. , Liang, C. , Li, Z. , Liu, Z. , Miao, B. , He, C. , & Sheng, L. (2015). Impact of precipitation patterns on biomass and species richness of annuals in a dry steppe. PLoS ONE, 10(4), e0125300 10.1371/journal.pone.0125300 25906187 PMC4407894

[ece34940-bib-0063] Yang, H. , Li, X. R. , Liu, L. C. , Gao, Y. H. , Li, G. , & Jia, R. L. (2014). Soil water repellency and influencing factors of Nitraria tangutorun nebkhas at different succession stages. Journal of Arid Land, 6(3), 300–310. 10.1007/s40333-013-0199-2

[ece34940-bib-0064] Yao, M. , Rui, J. , Niu, H. , Heděnec, P. , Li, J. , He, Z. , … Li, X. (2017). The differentiation of soil bacterial communities along a precipitation and temperature gradient in the eastern Inner Mongolia steppe. Catena, 152, 47–56. 10.1016/j.catena.2017.01.007

[ece34940-bib-0065] Zak, D. R. , Holmes, W. E. , White, D. C. , Peacock, A. D. , & Tilman, D. (2003). Plant diversity, soil microbial communities, and ecosystem function: Are there any links? Ecology, 84, 2042e2050 10.1890/02-0433

[ece34940-bib-0066] Zhang, Q. , Hou, X. Y. , Li, F. , Niu, J. , Zhou, Y. , Ding, Y. , … Kang, S. (2014). Alpha, beta and gamma diversity differ in response to precipitation in the Inner Mongolia grassland. PLoS ONE, 9(3), e93518 10.1371/journal.pone.0093518 24675900 PMC3968148

[ece34940-bib-0067] Zhang, Y. , Li, D. , Wang, H. , Xiao, Q. , & Liu, X. (2006). Molecular diversity of nitrogen‐fixing bacteria from the Tibetan Plateau, China. FEMS Microbiology Letters, 260, 134–142. 10.1111/j.1574-6968.2006.00317.x 16842336

[ece34940-bib-0068] Zheng, W. , Xue, D. , Li, X. , Deng, Y. , Rui, J. , Feng, K. , & Wang, Z. L. (2017). The responses and adaptations of microbial communities to salinity in farmland soils: A molecular ecological network analysis. Applied Soil Ecology, 120, 239–246. 10.1016/j.apsoil.2017.08.019

[ece34940-bib-0069] Zhong, Y. , Yan, W. , Wang, R. , Wang, W. , & Shangguan, Z. (2018). Decreased occurrence of carbon functions in microbial communities along with long‐term secondary succession. Soil Biology and Biochemistry, 123, 207–217. 10.1016/j.soilbio.2018.05.017

