# Supplementary material for: Differentiations of determinants for the community compositions of bacteria, fungi, and nitrogen fixers in various steppes
Source: Ecol Evol. 2019 Feb 22;9(6):3239–50. doi: 10.1002/ece3.4940 (PMC6434564; doi:10.1002/ece3.4940)
Supplement: Supplementary file 1 [file ECE3-9-3239-s001.docx]

**FIGURE S1** Map of the sampling sites drawn by Google Earth

D1-D5 represent 5 sampling sites of desert steppe, T1-T5 represent 5 sampling sites of typical steppe, M1-M5 represent 5 sampling sites of meadow steppe.

**FIGURE S2** Microbial communities that significantly differently distributed among steppes on order level. (a) Bacteria; (b) Fungi; (c) diazotrophs. Statistical significance was test at 0.05 level using SPSS.
