# Supplementary material for: Differentiations of determinants for the community compositions of bacteria, fungi, and nitrogen fixers in various steppes
Source: Ecol Evol. 2019 Feb 22;9(6):3239–50. doi: 10.1002/ece3.4940 (PMC6434564; doi:10.1002/ece3.4940)
Supplement: Supplementary file 2 [file ECE3-9-3239-s002.docx]

**TABLE S1** Information of sampling sites

| Steppe types | Sample | Sampling region | Latitude/longitude coordinates | Altitude (m) | Topography | Parent material | Dominant plant species | Plant coverage (%) |
| --- | --- | --- | --- | --- | --- | --- | --- | --- |
| Desert | D1 | Siziwang banner | N41゜49.925';  E111゜53.842' | 1447 | Hill | Granite | *Stipa klemenzii; Cleistogenes squarrosa; Caragana microphylla Lam* | 10 |
|  | D2 | Sonid right banner | N42゜47'21.99";  E112゜40'46.22" | 1099 | High plain | Granite | *Stipa klemenzii; Cleistogenes songorica* | 10 |
|  | D3 | Sonid right banner | N43゜10.317';  E112゜56.739' | 1039 | Hill | Granite | *Stipa klemenzii; Caragana microphylla Lam* | 15 |
|  | D4 | Sonid right banner | N43゜24.680';  E113゜07.275' | 1001 | Hill | Mesozoic mudstone | *Stipa klemenzii* | 20 |
|  | D5 | Abaga banner | N43゜57.072';  E114゜38.685' | 1109 | Hill | Granite | *Stipa klemenzii; Caragana microphylla Lam* | 40 |
| Typical | T1 | Abaga banner | N43゜53.996';  E115゜20.438' | 1166 | Hill | Granite | *Leymus chinensis; Stipa grandis* | 70 |
|  | T2 | West Ujimqin banner | N44゜25.738';  E117゜27.602' | 1151 | Hill | Granite | *Leymus chinensis; Stipa grandis* | 50 |
|  | T3 | West Ujimqin banner | N44゜50.291';  E117゜53.271' | 935 | Hill | Granite | *Leymus chinensis; Artemisia frigida* | 40 |
|  | T4 | Wulaga | N45゜58.978';  E119゜10.382' | 915 | Riverbed | Alluvium | *Leymus chinensis; Stipa grandis;* | 40 |
|  | T5 | Wulaga | N46゜07.542';  E119゜12.619' | 931 | Hill | Granite | *Leymus chinensis; Cleistogenes squarrosa* | 85 |
| Meadow | M1 | Hulunbuir | N49゜30.269';  E119゜47.364' | 638 | Hill | Loess | *Leymus chinensis; Stipa baicalensis* | 55 |
|  | M2 | Hulunbuir | N49゜19.213';  E119゜43.256' | 660 | Flat ground | Loess | *Leymus chinensis; Stipa baicalensis; Cleistogenes squarrosa* | 70 |
|  | M3 | Hulunbuir | N49゜18.130';  E119゜06.102' | 656 | Flat ground | Loess | *Leymus chinensis; Stipa baicalensis; Cleistogenes squarrosa* | 50 |
|  | M4 | Hulunbuir | N49゜27.104';  E118゜15.772' | 633 | Hill | Granite | *Leymus chinensis; Stipa baicalensis; Artemisia frigida; Potentilla acaulis* | 50 |
|  | M5 | Hulunbuir | N49゜26.842';  E118゜38.516' | 592 | Flat ground | Granite | *Leymus chinensis; Stipa baicalensis; Cleistogenes squarrosa* | 75 |

**TABLE S2** OTU numbers in different microbial groups and their proportions in total OTU number in each steppe

| Groups^a^ | | OTU numbers in each group ^b^ | | |  | The proportion of OTUs in different groups to the total OTU number in each steppe (%) ^c^ | | | | | | | | |
| --- | --- | --- | --- | --- | --- | --- | --- | --- | --- | --- | --- | --- | --- | --- |
|  |  | Bacteria | Fungi | N_2_ fixer |  | Bacteria | | | Fungi | | | N_2_ fixer | | |
|  |  |  |  |  |  | Desert | Typical | Meadow | Desert | Typical | Meadow | Desert | Typical | Meadow |
| Common | D&T&M | 1892 | 291 | 78 |  | 85.46 | 81.90 | 90.10 | 63.26 | 47.55 | 51.41 | 29.21 | 29.00 | 31.97 |
| Bi-shared | T&M | 133 | 188 | 56 |  |  | 5.76 | 6.33 |  | 30.72 | 33.22 |  | 20.82 | 22.95 |
|  | T&D | 277 | 119 | 104 |  | 12.51 | 11.99 |  | 25.87 | 19.44 |  | 38.95 | 38.66 |  |
|  | D&M | 24 | 26 | 11 |  | 1.08 |  | 1.14 | 5.65 |  | 4.59 | 4.12 |  | 4.51 |
| Unique | D | 21 | 24 | 74 |  | 0.95 |  |  | 5.22 |  |  | 27.72 |  |  |
|  | T | 8 | 14 | 31 |  |  | 0.35 |  |  | 2.29 |  |  | 11.52 |  |
|  | M | 51 | 61 | 99 |  |  |  | 2.43 |  |  | 10.78 |  |  | 40.57 |

^a^ Groups: Common: OTUs shared by all three steppe types; T&M: OTUs shared by typical and meadow steppes; T&D: OTUs shared by typical and meadow steppes; D&M: OTUs shared by desert and meadow steppes; D: unique OTUs only detected in desert steppes; T: unique OTUs only detected in typical steppes; M: unique OTUs only detected in meadow steppes.

^b^ OTU numbers for bacteria, fungi and N_2_ fixer in common, partial shared and unique groups.

^c^ The proportion of OTUs in different groups to the total OTU numbers for bacteria, fungi, N_2_ fixer in each steppe.

**TABLE S3** Pearson correlations between environmental factors and microbial community diversity

|  | Bacteria | |  | Fungi | |  | Diazotrophs | |
| --- | --- | --- | --- | --- | --- | --- | --- | --- |
|  | Pearson Correlation | P-value |  | Pearson Correlation | P-value |  | Pearson Correlation | P-value |
| WR | -0.338 | 0.217 |  | -0.483 | 0.068 |  | -0.427 | 0.113 |
| SOM | 0.000 | 0.999 |  | 0.130 | 0.644 |  | 0.132 | 0.639 |
| DOC | 0.047 | 0.867 |  | 0.140 | 0.619 |  | -0.247 | 0.374 |
| TK | 0.269 | 0.332 |  | 0.450 | 0.093 |  | 0.428 | 0.112 |
| AK | 0.025 | 0.928 |  | -0.125 | 0.657 |  | 0.128 | 0.651 |
| TP | -0.265 | 0.339 |  | -0.089 | 0.752 |  | 0.015 | 0.956 |
| AP | 0.057 | 0.840 |  | 0.078 | 0.783 |  | -0.049 | 0.864 |
| PH | 0.110 | 0.696 |  | -0.214 | 0.443 |  | -0.232 | 0.405 |
| MAT | -0.079 | 0.779 |  | -0.481 | 0.069 |  | -0.437 | 0.103 |
| MAP | 0.185 | 0.509 |  | 0.586 | 0.022* |  | 0.422 | 0.117 |
| PC | 0.000 | 0.999 |  | 0.380 | 0.162 |  | 0.274 | 0.323 |
| PR | 0.109 | 0.698 |  | 0.542 | 0.037* |  | 0.592 | 0.020* |
| PD | 0.086 | 0.760 |  | 0.519 | 0.048* |  | 0.595 | 0.019* |

WR: Water repellency cessation time; SOM: Soil organic matter; TN: Total nitrogen; AN: available nitrogen; DOC: dissolved organic carbon; TK: Total potassium; AK: Available potassium; TP: Total phosphorus; AP: Available phosphorus; MAT: Mean annual temperature; MAP: Mean annual precipitation; PC: Plant coverage; PR: Plant richness; PD: Plant beta-diversity

* represent significant differences (*p*< 0.05).
